# Intraoperative Ultrasound for Removal of a Fishbone Foreign Body Embedded in the Tongue

**DOI:** 10.1155/2024/8594673

**Published:** 2024-02-14

**Authors:** Ryan C. Daniel, Amr F. Hamour, Justin Cottrell, Trung Le, Kevin M. Higgins

**Affiliations:** ^1^Department of Otolaryngology–Head & Neck Surgery, Temerty Faculty of Medicine, University of Toronto, Toronto, ON, Canada; ^2^Sunnybrook Health Sciences Centre, Toronto, ON, Canada

## Abstract

**Background:**

Ultrasound (U/S) is a dynamic imaging modality with many applications in medicine. In Otolaryngology, U/S is used routinely in the clinic with several evolving applications intraoperatively. *Case Report*. A 53-year-old male presented to the emergency department with dysphagia, odynophagia, hoarseness, and sensation of foreign body after ingesting fish. A CT scan identified an approximately 2 cm horizontally-oriented foreign body consistent with a fishbone embedded in the left posterolateral tongue. Intraoperative U/S was used to localize and remove the fishbone without complications.

**Results:**

The patient recovered well after surgery and was discharged home on postoperative day 2. No residual foreign body was found on the repeat CT scan.

**Conclusion:**

Our case demonstrates the effectiveness of intraoperative U/S for removal of fishbone foreign bodies from the tongue and serves to inspire future applications of this modality in Otolaryngology.

## 1. Introduction

Ultrasound (U/S) is a dynamic, cost-effective imaging modality that enables many interactive applications in medicine. In the field of Otolaryngology, U/S is used for diagnosis and surveillance of soft tissue masses, image-guided biopsies, and monitoring of free flap anastomoses among other applications [1]. U/S is also starting to be used selectively in the operating room. Some of the applications include localization of masses in endocrine surgery and assessment of surgical margin clearance in head and neck oncology [2, 3]. Here, we present a case of intraoperative U/S to assist with localization and removal of a fishbone foreign body.

## 2. Case

**Figure 1:**
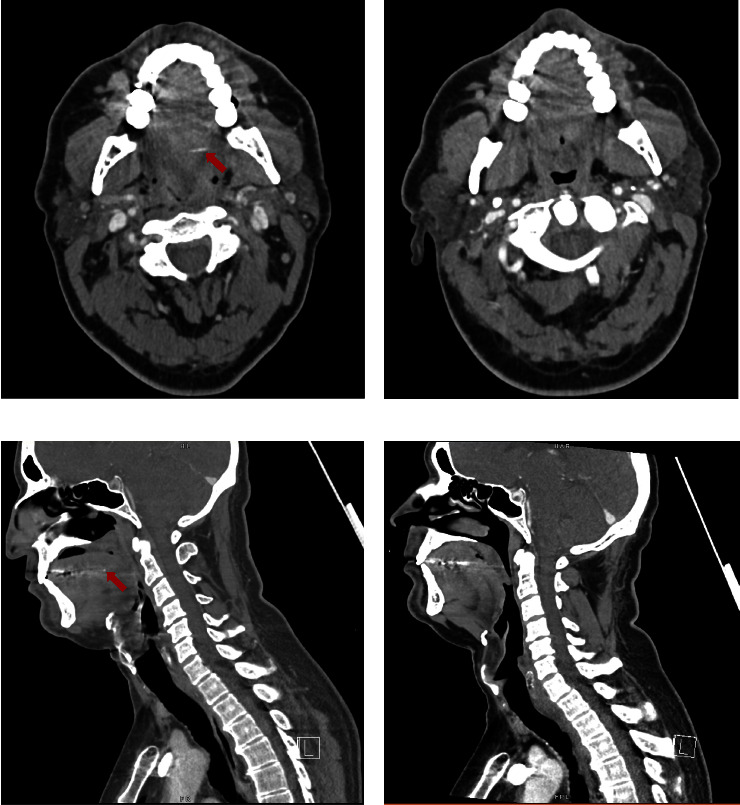
CT head and neck demonstrating the fishbone foreign body. (a) Representative preoperative axial image with a red arrow indicating the fishbone foreign body. (b) Representative postoperative axial image showing no residual foreign body. (c) Representative preoperative sagittal image with a red arrow indicating the fishbone foreign body. (d) Representative postoperative sagittal image showing no residual foreign body.

A 53-year-old male with hypothyroidism presented to the emergency department with dysphagia, odynophagia, hoarseness, and sensation of foreign body in his throat 2-3 hours after ingesting fish (Perch). On arrival, he was vitally stable and afebrile with no difficulty breathing or neck swelling. Basic blood work, including complete blood count, electrolytes, glucose, and creatinine, was largely unremarkable, with a white blood cell count of 4.1 (normal range: 4.0–11.0 × 10E9/L). Foreign body could not be identified on repeat clinical exams with palpation, soft tissue x-rays, or flexible nasolaryngoscopy by the Otolaryngology service. Given the patient's ongoing symptoms and the inability to localize the foreign body on initial investigations, a CT head and neck was ordered. The scan revealed an approximately 2 cm horizontal linear calcific density consistent with a fishbone in the left posterolateral tongue, at the junction of the oral and base of tongue between the intrinsic and extrinsic musculature. There was accompanying soft tissue swelling with no identifiable entry or exit site (Figures 1(a) and 1(c)). The patient was kept NPO, and maintenance of IV fluids, IV dexamethasone (6 mg IV q8h), IV cefazolin/metronidazole (1 g q8h; 500 mg q12h), and prn analgesia (acetaminophen/ketorolac) were initiated. After thorough discussion between the patient and surgical team, it was decided that operative management was indicated given the organic nature of the impaction and the potential for abscess formation and possible airway compromise.

**Figure 2:**
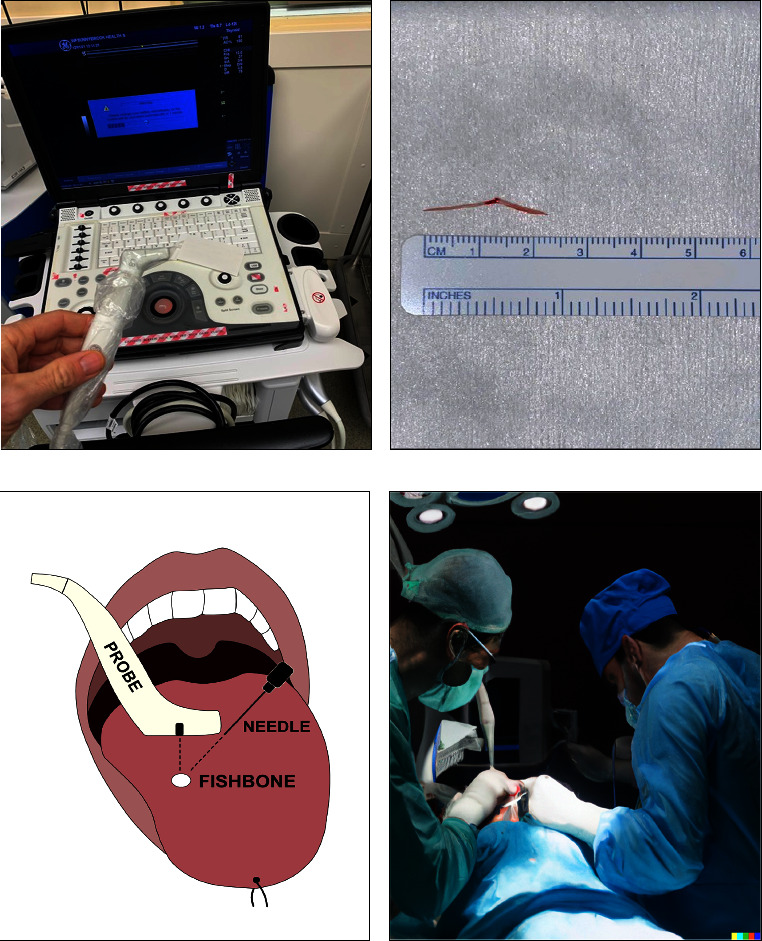
Details of the intraoperative U/S guided procedure. (a) GE linear hockey stick U/S probe. (b) Photograph of the removed fishbone. (c) Cartoon depiction of the intraoperative fishbone foreign body localization using 18-gauge needle triangulation under ultrasound guidance. (d) Computer-generated rendering depicting the intraoperative U/S guided procedure.

The patient was brought to the operating theatre within 24 hours of the CT scan findings. Following nasotracheal intubation with general anesthesia, a bite block was placed and the entire oral cavity was thoroughly inspected. A retraction suture was placed through the tongue tip to deliver the tongue and facilitate access. Intraoral examination and direct laryngoscopy revealed only mild erythema of the left posterolateral tongue without any clear point of entry. A longitudinal 1.5 cm incision was made through the midline raphe to the transition of the intrinsic and extrinsic tongue musculature to ensure that an avascular plane was maintained. The fishbone could not be identified following initial surgical exploration based on preoperative CT triangulation. To better localize the foreign body, a “hockey stick” small footprint linear array U/S transducer (General Electric Healthcare; L8-18i-D) was brought into the operating room (Figure 2(a)). Using the transducer, a hyperechoic object compatible with a fishbone was identified 1.2 cm deep, sitting horizontally anterior to the right tongue base and crossing midline. An 18-gauge, 3.8 cm long needle, was used to triangulate further on the lesion in combination with U/S guidance. Care was taken to remain in an avascular plane and avoid the dorsal lingual artery. The fishbone was identified by following the tract of the needle and it was removed in its entirety (Figures 2(b)–2(d)). Good hemostasis was achieved, and the wound was left to heal by secondary intention.

Postoperatively, the patient was kept intubated and transferred to the ICU for monitoring while remaining on IV dexamethasone and IV cefazolin/metronidazole. They were extubated the following morning without any complications and transferred to the general ward. Once extubated, the patient's diet was slowly advanced from clear fluids to a soft foods. A repeat CT scan on postoperative day 1 showed no residual foreign body (Figures 1(b) and 1(d)). IV dexamethasone was stopped 24 hours after surgery. There were no immediate postoperative complications, and the patient was discharged home on postoperative day 2 in stable condition. They were instructed to continue with a soft diet for several weeks, complete a 7-day course of oral antibiotics with clindamycin 450 mg three times daily, and continue chlorohexidine gluconate 0.12% oral rinses 10 mL four times daily for 7–10 days. They recovered well without any long-term complications and the tongue completed granulation after several weeks.

## 3. Discussion

Foreign bodies account for a significant portion of emergency cases seen in Otolaryngology, estimateed between ∼10% and 30% of all cases depending on the centre. They occur more frequently in younger children but can spontaneously occur in both pediatric and adult populations [4]. The potential short-term sequela without prompt treatment includes perforation,neurovascular injury and airway compromise, while long-term, patients can develop chronic pain, abscesses, granulomas, and migration that can damage surrounding structures [5–8]. Therefore, locating and removing foreign bodies on the initial assessment is vital. Inflammation, the relative size of the embedded object, poor visualization, and exposure can make identifying foreign bodies embedded deep within soft tissue structures, like the tongue, very challenging with physical exam alone. As a result, identification is often missed in acute care settings and retained foreign bodies are among the top three causes for litigation after wound care [9, 10]. Our case illustrates this challenge, given that the fishbone could not be located with only physical examinations and scopes by the Emergency Medicine and Otolaryngology service.

Radiologic investigation enhances the ability to identify foreign bodies embedded within soft tissue structures. Plain radiographs, MRI, CT, and U/S can all be used with unique strengths and limitations. Plain radiographs are most commonly used given their widespread availability, but they lack sensitivity for foreign bodies with compositions similar to soft tissues, such as wood and plastic. MRI can be used to detect radiopaque and radiolucent objects, but it is infrequently used due to cost, availability, and safety risk if there is any potential that the foreign body is ferromagnetic. CT is widely considered the gold standard for detecting foreign bodies. Similar to plain radiographs, it is very sensitive for detecting radiopaque objects, with improved detection of radiolucent objects due to summation effects. The 3D reconstruction of CT scans also assists with determining the depth of penetration and impact on surrounding structures. With that said, CT carries a risk of radiation, and in patients with metallic implants, metal-related artefacts can significantly impair image quality. U/S is a relatively new imaging modality for detecting foreign bodies. It is widely available and cost-effective, without any risk of radiation. In comparison to CT and plan radiographs, it also may be more reliable for detecting radiolucent objects and offers the unique advantage of dynamic imaging that is ideal for procedures [11]. The main drawback is that structures surrounded by air or bone are difficult to see due to attenuation of the U/S waves [12]. The migrating nature and poor palpation characteristics of a fishbone, in addition to the challenges of identifying a foreign body surgically within the tongue musculature, makes blind surgical exploration a more morbid approach with decreased success of retrieval. It is worth noting in this case, that real-time dynamic imaging was crucial, as the fishbone had migrated from its original left-sided position noted on CT, to the right side, and initial surgical exploration without U/S guidance was not able to localize the foreign body.

For foreign bodies embedded in the musculature of the tongue, our experience supports the use of CT for the initial assessment followed by U/S for dynamic localization and removal intraoperatively. We used a GE “hockey stick” probe, which was ideal for this application given its small size, allowing it to fit inside the oral cavity, and high frequency, which enabled the production of dynamic high-resolution images across its linear footprint. Several manufacturers, including GE (L8-18i-D), Phillips (L15-7io), Samsung (LS6-15), and BK Medical (8809), have designed their own versions of the “hockey stick” probe that are compatible with their corresponding clinical grade machines. Lower frequency probes, such as the curvilinear or abdominal probe and the classic linear probe, would not be as effective due to their inability to fit in the oral cavity and relatively poor resolution at the depth of soft tissues. Given that U/S is used routinely by Otolaryngologists, the hockey stick probe can easily be purchased and added to their arsenal for intraoperative foreign body removal.

## 4. Conclusion

In conclusion, our case is a unique example of foreign body ingestion requiring surgical intervention in an individual without obvious risk factors. We have found only three previous reports that discussed the use of intraoperative U/S to localize and remove aerodigestive foreign bodies, which included two fishbones and an airgun pellet [13–15]. We used a similar technique to that described by Smith et al. where U/S was used in conjunction with a needle for triangulation and removal of a fishbone [13]. Two additional reports have described the use of U/S to localize and remove aerodigestive foreign bodies in the emergency department [16, 17]. This case adds to the growing literature supporting new uses of U/S for dynamic intraoperative imaging in Otolaryngology.

## Data Availability

The data is not publicly available due to privacy and ethical restrictions.
